# Long-Term Co-Circulation of Host-Specialist and Host-Generalist Lineages of Group B *Streptococcus* in Brazilian Dairy Cattle with Heterogeneous Antimicrobial Resistance Profiles

**DOI:** 10.3390/antibiotics13050389

**Published:** 2024-04-25

**Authors:** Laura Maria Andrade de Oliveira, Leandro Correia Simões, Chiara Crestani, Natália Silva Costa, José Carlos de Figueiredo Pantoja, Renata Fernandes Rabello, Lucia Martins Teixeira, Uzma Basit Khan, Stephen Bentley, Dorota Jamrozy, Tatiana de Castro Abreu Pinto, Ruth N. Zadoks

**Affiliations:** 1 Instituto de Microbiologia Paulo de Góes, Universidade Federal do Rio de Janeiro, Rio de Janeiro 21941-902, Brazil; leandrosimoes@micro.ufrj.br (L.C.S.); nataliacosta@micro.ufrj.br (N.S.C.); lmt2@micro.ufrj.br (L.M.T.); tcap@micro.ufrj.br (T.d.C.A.P.); 2 Institut Pasteur, 75015 Paris, France; chiara.crestani@pasteur.fr; 3 Faculdade de Medicina Veterinária e Zootecnia, Universidade Estadual Paulista Júlio de Mesquita Filho, Botucatu 18618-681, Brazil; jose.pantoja@unesp.br; 4 Instituto Biomédico, Universidade Federal Fluminense, Niterói 24210-130, Brazil; rerabello@id.uff.br; 5 Wellcome Trust Sanger Institute, Hinxton, Cambridge CB10 1SA, UK; uk1@sanger.ac.uk (U.B.K.); sdb@sanger.ac.uk (S.B.); dj9@sanger.ac.uk (D.J.); 6 Sydney School of Veterinary Science, Faculty of Science, University of Sydney, Camden, NSW 2570, Australia

**Keywords:** group B *Streptococcus*, antimicrobial resistance, molecular epidemiology, bovine mastitis, dairy cattle

## Abstract

Group B *Streptococcus* (GBS) is a major cause of contagious bovine mastitis (CBM) in Brazil. The GBS population is composed of host-generalist and host-specialist lineages, which may differ in antimicrobial resistance (AMR) and zoonotic potential, and the surveillance of bovine GBS is crucial to developing effective CBM control and prevention measures. Here, we investigated bovine GBS isolates (*n* = 156) collected in Brazil between 1987 and 2021 using phenotypic testing and whole-genome sequencing to uncover the molecular epidemiology of bovine GBS. Clonal complex (CC) 61/67 was the predominant clade in the 20th century; however, it was replaced by CC91, with which it shares a most common recent ancestor, in the 21st century, despite the higher prevalence of AMR in CC61/67 than in CC91, and high selection pressure for AMR from indiscriminate antimicrobial use in the Brazilian dairy industry. CC103 also emerged as a dominant CC in the 21st century, and a considerable proportion of herds had two or more GBS strains, suggesting poor biosecurity and within-herd evolution due to the chronic nature of CBM problems. The majority of bovine GBS belonged to serotype Ia or III, which was strongly correlated with CCs. Ninety-three isolates were resistant to tetracycline (≥8 μg/mL; *tet*O = 57, *tet*M = 34 or both = 2) and forty-four were resistant to erythromycin (2.0 to >4 μg/mL; *erm*A = 1, *erm*B = 38, mechanism unidentified *n* = 5). Only three isolates were non-susceptible to penicillin (≥8.0 μg/mL), providing opportunities for improved antimicrobial stewardship through the use of narrow-spectrum antimicrobials for the treatment of dairy cattle. The common bovine GBS clades detected in this study have rarely been reported in humans, suggesting limited risk of interspecies transmission of GBS in Brazil. This study provides new data to support improvements to CBM and AMR control, bovine GBS vaccine design, and the management of public health risks posed by bovine GBS in Brazil.

## 1. Introduction

*Streptococcus agalactiae* or Group B *Streptococcus* (GBS) is a versatile multi-host pathogen that can cause infections in humans and animals [1,2,3]. GBS is a major cause of neonatal meningitis and sepsis worldwide, and can cause stillbirths, preterm births, newborn deaths, and long-term disabilities in affected babies [4]. GBS can colonize the human gastrointestinal and genital tracts asymptomatically, and maternal rectovaginal colonization during pregnancy is the main source of GBS transmission to newborns [1]. In addition, GBS can cause diseases in pregnant women [1,5] and non-pregnant adults [6], including zoonotic and foodborne invasive GBS [7]. In animals, GBS is a leading cause of streptococcosis in fish [2] and of contagious bovine mastitis (CBM) [3,8]. In dairy cattle, the mammary gland is the main reservoir of GBS, and the bacteria are primarily transmitted directly from cow to cow during the milking process [9]. In addition, the occurrence of bi-directional interspecies transmission between people and cattle is supported by genetic, genomic, and evolutionary data [10,11], with transmission pathways including direct contact and the consumption of milk [12]. In high-income countries, CBM due to GBS has largely been controlled with industry-wide mastitis-management programs [11,13]. This is not the case, however, in low- and middle-income countries (LMIC) or in emerging economies such as China or Brazil.

Brazil is among the top 10 milk-producing countries globally, but its dairy industry suffers economic losses from CBM [14]. CBM control in Brazil is challenging because of the heterogeneous nature of the dairy industry, in which nearly 60% of dairies are family farms, and because of antimicrobial misuse in dairy herds, including the use of broad-spectrum products and Highest Priority Critically Important Antimicrobials, which include third- and fourth-generation cephalosporins, fluoroquinolones, polymyxins, and macrolides [10,14]. Macrolide (e.g., erythromycin) and lincosamide (e.g., clindamycin) resistance is common in human [15,16] and bovine [15,17] GBS in large parts of the world, and has been classified as a “concerning threat” [18]. In countries where industry-wide udder health management programs for dairy farms are poorly developed and where the use of antimicrobials is indiscriminate, prevention of the spread of GBS and antimicrobial resistance (AMR) through vaccination is highly desirable [19]. In humans, promising maternal GBS vaccines based on bacterial subunits (e.g., capsule, surface proteins) are undergoing clinical trials [4], and GBS vaccines are commercially available for fish; however, the same progress has not yet been achieved for a bovine GBS vaccine [20].

As a bacterial species, GBS is composed of host-generalist lineages such as clonal complex (CC) 10, which is found in people, cattle, and fish, and host-specialist lineages (e.g., CC17 as a cause of human neonatal disease and CC61 as a cause of mastitis in dairy cattle), which may differ in virulence, AMR, zoonotic potential, and immunogenicity [1,2,3]. The prevalence of host-generalist and host-specialist GBS lineages in dairy cattle varies between countries and continents, with a predominance of host-specialist GBS in some European countries, e.g., Portugal and the UK [21,22], and a mixture of host-specialists and host-generalists in other European countries, e.g., the Nordic countries, and in Colombia [8,12]. The lack of data regarding the molecular epidemiology of bovine GBS, especially in LMIC and emerging economies, impairs the development of a universally effective vaccine to prevent CBM due to GBS [20]. In addition to informing vaccine design, characterization of the GBS population structure can uncover modes of pathogen transmission and identify AMR strains of concern [8,11].

In this study, we investigated a collection of historical and contemporary bovine GBS isolates collected in Brazil over a 35-year period using phenotypic testing and short-read whole-genome sequencing to uncover the molecular epidemiology of mastitis-causing GBS strains in one of the world’s largest dairy industries. Our work will help raise awareness of the potential public health risks posed by bovine GBS, tackle the spread of AMR, improve CBM control, and inform bovine GBS vaccine design.

## 2. Results

### 2.1. Descriptive Analysis

**Figure 1:**
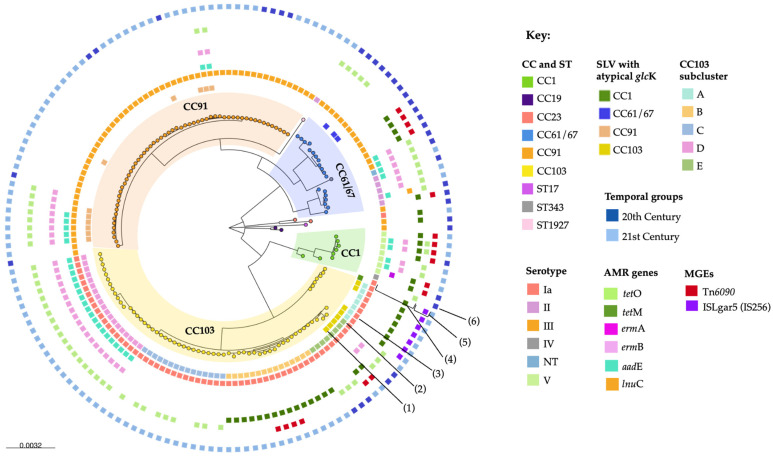
Core gene phylogeny of 156 historical (1987–2000) and contemporary (2001–2021) bovine Group B *Streptococcus* isolates from 45 herds in Brazil. The tree was rooted at the midpoint. Leaf nodes are colored according to the clonal complexes (CCs) or sequence types (STs). Branches of the tree are highlighted to indicate clades representing the dominant CCs. Metadata are shown from the innermost to the outermost circle, as follows: (1) single-locus variant (SLV) with atypical *glc*K gene, (2) CC103 subclusters, (3) serotype, (4) *aad*E, *erm*A, *erm*B, *lnu*C, *tet*M, *tet*O, (5) Tn*6090*, ISLgar5 family IS256, and (6) temporal groups. AMR: antimicrobial resistance; MGEs: mobile genetic elements.

All GBS isolates had high MALDI-ToF MS scores for species identity (>2.2) and showed a CAMP (Christie–Atkins–Munch-Peterson) reaction and hippurate hydrolysis. In total, 5 (3.2%) GBS isolates were negative for lactose fermentation (*n* = 4 from milk and *n* = 1 environmental) and 151 (96.8%) isolates were positive. Across the 156 isolates from 45 herds, 5 serotypes and 18 sequence types (STs) were detected. Bovine GBS isolates were predominantly distributed across four clades, occurring in two pairs with a common ancestor per pair. Approximately half of all isolates were included in a pair of clades formed of CC61/67 and CC91 (Figure 1). Most of the remaining isolates formed another pair of clades that included CC1 and CC103, with a handful of isolates from CC17, CC19, and CC23 forming minor clusters (Figure 1). We could not determine the STs of 24 GBS isolates. Those isolates showed atypical *glcK* sequences due to an indel event that was split across two contigs, and it was not possible to assign an allele number to them using the PubMLST database (https://pubmlst.org/, accessed on 15 November 2023). Based on the combination of the remaining six alleles, isolates with atypical *glcK* sequences belonged to multiple CCs, including CC1, CC61/67, CC91, and CC103, and originated from multiple herds (Table S1). To obtain *glcK* sequence data, a PCR reaction targeting the *glcK* gene was performed, which produced amplicons of approximately 1000 bp, more than double the size of a normal amplicon (459 bp) [23]. The amplicons were sequenced by the Sanger sequencing method, and high-quality sequences were obtained for four fragments from isolates belonging to CC91 or CC103. A consensus sequence was obtained with Geneious Prime v.2023.2, showing that *glcK* was truncated from position 408 until the end of the reference *glcK* sequence.

### 2.2. Within-Herd Analysis

Because GBS is largely spread by cow to cow transmission, the composition of the isolate population may be skewed by the overrepresentation of herds where multiple cows were sampled. To correct for this, we conducted within-herd analysis prior to between-herd analysis, following the example of Lyhs and colleague [12]. Among 21 herds with multiple isolates, isolates could belong to one or multiple CCs and to one or multiple strains within a CC, whereby strains are defined as “an isolate or group of isolates that can be distinguished from other isolates of the same genus and species by phenotypic and genotypic characteristics” [24]. Here, we used the combination of sequence type (ST), serotype, AMR genes, and mobile genetic elements (MGEs) to define strains. Complete strain homogeneity was observed in seven herds with 2 to 11 isolates per herd (Table S1). The presence of a single CC but with heterogeneity in ST, AMR, or MGE patterns was observed in seven herds with 1 to 11 isolates per strain per herd. For example, ST91 and ST103 each co-circulated with their respective SLV with atypical *glcK* in several herds, and CC1 isolates carrying *aad*E, *erm*B, and *tet*O occurred with or without transposon Tn*6009* and *tet*M within herds (Figure 1, Table S1). The presence of two CCs per herd without heterogeneity within either CC was observed in three herds, with 1 to 11 isolates per CC per herd. Finally, the presence of multiple CCs as well as strain heterogeneity at sub-CC level was observed in four herds, with 1 to 9 isolates per strain per herd (Table S1). The co-circulation of strains that belonged to different CCs and serotypes, and even to separate clades in the phylogenetic tree, occurred among historical and contemporary isolates. For example, ST103/serotype Ia co-circulated with ST1930/serotype III in 1999, with ST19/serotype III in 2010, and with ST91/serotype III in 2004, 2015, and 2021 (Table S1). Based on the combination of ST, serotype, AMR genes, and MGE, 67 unique herd–strain combinations were identified, and these were included in the between-herd analysis.

### 2.3. Between-Herd and Temporal Analyses

**Figure 2:**
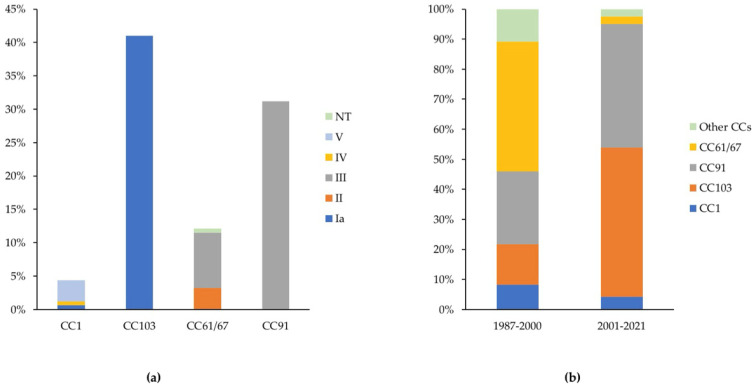
Between-herd analysis of the bovine Group B *Streptococcus* (GBS) population (*n* = 67 strain–herd combinations from 45 herds) in Southeastern Brazil showing (**a**) the association of CCs with serotypes and (**b**) a temporal shift in predominant clonal complexes (CCs). NT = non typable.

Clonal complexes (CCs) 103/serotype Ia and CC91/serotype III made up the majority of the GBS population as defined by unique herd–strain combinations (21/67 or 31.3% and 19/67 or 28.4%, respectively). Other CCs and serotypes were less common and included CC61/67 (12/67, 18%; three serotypes, with III as the dominant type), CC1 (8/67, 12%; three serotypes, with V as the dominant type), CC19 (2/67, 3%), and CC23 (2/67, 3%) (Figure 2a, Table S1). Among the historical herd–strain combinations (1987–2000; *n* = 25), CC61/67 was most common (10/25 or 40%), followed by CC103 (5/25, 20%) and CC91 (3/25, 12%). By contrast, among the contemporary herd–strain combinations (2001–2021, *n* = 42), CC103 and CC91 predominated (16/42 or 38% each) and CC61/67 was rarely detected (2/42 or 5%). The prevalence of CC1 remained stable (12% among the historical and contemporary herd–strain combinations), and the remaining strains were detected at low frequencies (Figure 2b; Table S1).

The CC103 isolates representing unique herd–strain combinations belonged to ST103 (*n* = 12), ST314 (*n* = 1), or single-locus variants (SLV, *n* = 8) and were first detected in 1997, while the CC91 isolates belonged to ST91 (*n* = 10), ST1934 (*n* = 2), or SLV (*n* = 7), and their first detection in our collection dates from 1999 (Supplementary Figure S1). CC61/67 was formed of ST67 (*n* = 4), ST61 (*n* = 1), or SLVs (*n* = 7); CC1 encompassed ST1 (*n* = 6) and SLVs (*n* = 2); CC19 included only ST19 isolates (*n* = 2); and CC23 was formed of ST23 (*n* = 1) and ST55 (*n* = 1). Additionally, we detected ST17 and ST343 as singletons and identified six new STs. The three new STs that belonged to CC61/67 (ST1929, ST1930, ST1932) were found among historical isolates only (1998, 1999, and 1996, respectively), whereas the three new STs that did not belong to CC61/67 were found among recent isolates only, i.e., ST1918 (CC103) in 2015 and ST1927 (singleton) as well as ST1934 (CC91) in 2021.

### 2.4. Antimicrobial Resistance

One hundred and one GBS isolates harbored at least one AMR gene based on genomic analysis (Table S1) and were selected for phenotypic AMR testing because resource constraints precluded testing of the entire collection. Of the 101 isolates tested, 93 were phenotypically resistant to tetracycline (≥8 μg/mL) and carried *tet*O (*n* = 57), *tet*M (*n* = 34), or both (*n* = 2). Three isolates with tetracycline resistance genes, one carrying *tet*M and two carrying *tet*O, were phenotypically susceptible to tetracycline, indicating imperfect agreement between the genotype and phenotype. Forty-four isolates were resistant to erythromycin (2.0 to >4 μg/mL; *erm*A *n* = 1, *erm*B *n* = 38, mechanism unidentified *n* = 5), while three *erm*B-positive isolates were phenotypically susceptible. Forty-three isolates were phenotypically resistant to pirlimycin (≥4 μg/mL; *erm*B *n* = 38, mechanism unidentified *n* = 5). The lincosamide resistance gene *lnu*C was detected in one isolate but it was phenotypically susceptible.

Using the Centers for Disease Control and Prevention (CLSI) criteria for β-hemolitic *Streptococcus* spp., where macrolides and lincosamides are classed as different categories [25], we found that 42 isolates from 16 herds were phenotypically resistant to three antimicrobial categories (tetracycline, macrolide, lincosamide), and hence, were classed as multidrug-resistant (MDR). This included two isolates originating from two herds that were also non-susceptible to penicillin/novobiocin (≥8/16 μg/mL), three isolates from two herds that were non-susceptible to penicillin (≥8.0 μg/mL), and two isolates from two herds that were resistant to ampicillin (>8.0 μg/mL). The remaining isolates were susceptible to the narrow-spectrum beta-lactams (penicillin, ampicillin) and cephalosporins (ceftiofur, cephalothin) that were tested. Thirteen GBS isolates showed sulfadimethoxine MICs of 64.0 to >256, but there is no breakpoint definition for this drug [25,26].

The association between AMR gene prevalence, time periods, and population structure was explored across the 67 herd–strain combinations. The prevalence of resistance was not associated with the time of collection (historical vs. recent, Chi-square, df = 1, *n* > 0.27 for each gene or for overall AMR gene prevalence), but there was an association with CCs (Figure 1). Most isolates from CC91 were susceptible to all antimicrobials tested. Three isolates in CC91, originating from three herds (H10, H14, and H4) and three years (2015, 2020, and 2021, respectively), all representing the *glcK* variant of ST91, carried the *tet*O, *erm*B, and *aad*E genes. In one of those herds, two strains carried *tet*O and *erm*B only, belonging to ST91 or its *glcK* variant SLV. By contrast, AMR was common in CC103, and the prevalence and combinations of AMR genes were associated with subclusters within CC103 (Figure 1). Isolates in the CC103 subcluster A (*n* = 7) were obtained from four herds in 2007, and harbored *tet*M and insertion sequence ISLgar5 (IS*256* family). All CC103 subcluster B isolates (*n* = 15; 2001, 2015, 2021; herds *n* = 2) harbored *tet*M, and three isolates also carried transposon Tn*6009*; CC103 subcluster C comprised fifteen isolates from a single herd in 2004, of which nine carried *tet*O; and CC103 subcluster D included nineteen isolates from three herds sampled in 2002, 2010, and 2015, respectively, all of which harbored the *aad*E, *erm*B, and *tet*O genes. Finally, CC103 subcluster E, which included historical (1997 and 1999) and recent (2000, 2001, 2004, and 2006) isolates from eight different herds, was associated with a variety of AMR and MGE profiles, including *tet*O only (*n* = 4), *tet*O and *erm*B (*n* = 2), or *tet*M and Tn*6009* (*n* = 2) (Figure 1). Like CC103 subcluster E, CC61/67 included numerous AMR and MGE profiles, including MDR isolates based on the presence of *aad*E, *erm*B, and *tet*O (three herds sampled in 1999, 2000, and 2001) or *aad*E, *lnu*C, and *tet*O (one herd sampled in 1996).

## 3. Discussion

In this study, we used a unique collection of historical and contemporary bovine GBS isolates recovered in Brazil over more than three decades to explore the molecular epidemiology of mastitis-causing GBS and determine the need for public health measures and priorities for CBM management and prevention. The most prevalent lineages were CC61/67, CC91, and CC103, none of which are common GBS strains in human disease. A similar predominance of bovine-associated lineages has been reported in Portugal [21] and China [3]. This is in contrast to several Northern European countries and Colombia, where GBS from mastitis was associated with lineages also found in humans, notably CC1 and CC23 [8,9,12]. For the latter, interspecies transmission is strongly suspected [8,27], and carriage as well as disease has been reported in humans [12]. Although the major mastitis-causing GBS lineages found in Brazil are not commonly associated with human disease, they may act as a reservoir of AMR, a concern that is heightened because of the often indiscriminate use of antimicrobials in Brazil.

Resistance to tetracycline was widespread in bovine GBS from Brazil. Approximately 70% of herd–strain combinations carried *tet*M, which is the most common human-associated tetR gene [28], or *tet*O, which was found to be the most common bovine-associated tetR gene in a study in the USA [29]. Macrolide, lincosamide, and streptogramin B (MLS_B_) resistance genes were found in nearly a third of herd–strain combinations. A high prevalence of *tet*M, *tet*O, *erm*B, and *erm*A has been reported in other emerging dairy industries and economies, such as Colombia [8], Argentina [30], and China [31,32], ranging from 9% to 74% for tetR and from 14 to 74% for MLS_B_ resistance genes. We also detected the aminoglycoside resistance gene *aadE*. Resistance to aminoglycosides mediated by *aad*E has been reported for human clinical GBS in the UK [33], South Korea [34], and China [35], and for bovine mastitis isolates belonging to the species *Staphylococcus aureus* [36]. To our knowledge, it has not been described in bovine GBS previously. The fact that *S. aureus* and GBS both cause CBM and may co-circulate within dairy herds due to shared biological and epidemiological drivers of transmission raises the possibility of the interspecies transmission of *aad*E. Aminoglycoside resistance in GBS can also be encoded by *aph*A or *aad6*, as reported in Argentina (*aph*A) [28] and China (*aph*A and *aad6*) [37]. 

Considering the widespread and often inappropriate use of antimicrobials in the Brazilian dairy industry, we had anticipated an increase in AMR prevalence in contemporary isolates compared to historical isolates, but this was not observed. Indeed, the bovine-adapted lineage CC61/67, which was common among historical isolates and often carried one or more AMR genes, was largely replaced by another bovine-adapted lineage, CC91, which rarely carries AMR genes (Figure 1). CC91 shares its most recent common ancestor with CC61/67 and appears to represent a regional adaptation, as previously reported in other Southeast and Northeast regions of Brazil [38]. The evolutionary driver of this adaptation, which favored the expansion of the largely susceptible clade CC91 over the commonly resistant clade CC61/67, despite the selective pressure exerted by antimicrobial use, is unknown. The acquisition of AMR genes by bacterial pathogens may impose a fitness cost through the dysregulation of gene expression and alteration in important physiologic processes, depending on their mechanism of action, e.g., target alteration, as shown for *Mycobacterium tuberculosis* [39]. A similar phenomenon could potentially have contributed to the replacement of CC61/67 by CC91. In contrast to CC91, almost all CC103 isolates carried AMR genes. CC103 in our collection was first isolated in 1997, followed by an increase in its prevalence among more recent isolates. The emergence of CC103 has been described in countries with high antimicrobial use, e.g., Colombia [8] and China [3], but also in countries with very restrictive antimicrobial use policies, e.g., Denmark, Sweden, and Finland [9,11,12]. This emergence may be driven, at least in part, by differences in sources and transmission routes between CC103 and other bovine GBS, with CC103 possibly disseminated via the environment rather than by contagious transmission [40].

In contrast to what has been reported from previous studies [9], our analysis showed considerable strain heterogeneity within herds, including the co-circulation of GBS lineages with distinct CC, ST, AMR, or MGE patterns. Both at the core genome and at the accessory genome level, heterogeneity was observed in multiple herds, showing that the biological or evolutionary processes leading to heterogeneity occur repeatedly and pose an ongoing risk for the emergence of new strains. The co-circulation of distinct CCs has also been described in Colombia [8] and China [3] and suggests repeated introductions due to poor biosecurity. The operation of closed herds, or the screening of animals for GBS prior to their introduction to a herd, could reduce this risk. Ideally, a point-of-care test would be available for this purpose, as access to diagnostic laboratories for milk testing is limited in Brazil. In addition to repeated introductions, there is evidence for within-herd evolution of the core genome, as shown by the co-circulation of ST67, ST91, and ST103 and their respective SLVs. Within-herd evolution, including the development of SLVs or variants that can be detected with other molecular methods, is likely to occur as the number of bacterial replication cycles increases, e.g., due to CBM problems that last many years or affect a large number of cows [41]. To control this, improved GBS detection and control are required, e.g., the use of bulk milk screening for the early detection of GBS at herd level, the use of individual cow milk somatic cell counts to select cows for bacterial culture, the treatment of GBS-positive cows, the culling of cows with chronic infection or treatment failure, the prevention of transmission during milking, and the application of post-milking teat disinfectants [42]. Importantly, from an antimicrobial stewardship perspective, almost all isolates in our study were susceptible to penicillin, and the intramammary application of narrow-spectrum penicillin is the treatment of choice for GBS mastitis [42].

In highly developed dairy industries, the control measures outlined above have largely been sufficient to control CBM caused by GBS [11,13], but in emerging dairy industries, additional control tools such as vaccines may be desirable. For the prevention of GBS infections in humans, the use of a hexavalent vaccine has been recommended, with serotype III as the most important serotype [43]. Among our bovine GBS isolates, serotypes Ia and III were the most common. For the major clades, there was a clear association between CCs and serotypes (CC91/serotype III, CC103/serotype Ia) whereas other CCs were associated with multiple serotypes, e.g., serotypes Ia, IV, and V, were found among CC1 isolates. Other CCs commonly encompass multiple serotypes, e.g., serotypes II and III were common among CC61/67 isolates in our study, whilst serotype Ia and non-typeable isolates have also been reported for CC61/67 [3,22,38]. Such heterogeneity may be due to capsular serotype switching, which has been described in human [44,45] as well as bovine GBS [11]. Two serotypes represented the majority of bovine GBS isolates in the current study, providing a potential focus for vaccine development, but minority serotypes (serotype II in CC61/67, serotypes Ia and IV in CC1) were associated with MDR genotypes. Thus, it would probably be wise to include all four serotypes in a bovine GBS vaccine.

Twenty-four GBS isolates showed atypical *glc*K alleles due to an indel event in this locus. The BLAST alignment showed the atypical *glc*K sequence aligned with two atypical GBS *glc*K genes with the insertion of a 2000 bp MGE related to group II introns. Those atypical *glc*K genes were detected in the genomes of human clinical and carriage strains that belonged to serotypes II and V [46,47]. Partial deletion of the *glcK* locus has been described in GBS from fish in Brazil [48]. The fish isolates were classified as an SLV of ST257, and form part of a uniquely fish-associated clade [49]. In our bovine GBS study, the atypical *glc*K allele was found across the major CCs identified in the GBS population, CC1, CC61/67, CC91, and CC103, raising questions about the classification of *glc*K as a housekeeping gene in GBS. Further studies at population and mechanistic levels are needed to provide a better understanding of the functional relevance of the atypical *glc*K genes in GBS across host species.

The limitations of this study include the geographical scope (we analyzed GBS strains from herds located in only one of the five macroregions of Brazil (the Southeast region), the opportunistic nature of the sample and isolate collection (for example, we did not have GBS strains representative of all years within the timeline investigated (1987 to 2021)), and the lack of herd-specific management data. Even so, we can observe clear changes in the composition of the GBS population and provide suggestions for further research and improved herd management and antimicrobial stewardship.

## 4. Materials and Methods

### 4.1. Bacterial Isolates

The GBS isolates (*n* = 156) analyzed in this study were collected over a period of more than three decades (1987 to 2021, inclusive) in the Southeast region of Brazil and stored in Difco™Skim Milk (Becton Dickinson, Franklin Lakes, NJ, USA) at −20 °C as part of our culture collection. Most isolates originated from bovine milk (*n* = 151), with the remainder originating from a dairy farm environment (milking machine, cleaning water, sand; *n* = 5). From 24 herds, a single isolate was available, and from 21 herds, multiple isolates were available (*n* = 2 to 17, median = 5). Isolates were divided into “historical” (1987–2000; *n* = 37), and “recent” (2001–2021; *n* = 119) categories for comparative purposes. All GBS isolates included in this study were recovered during epidemiological surveillance studies, which do not require ethics approval, or sent to our laboratory by research institutions, as approved by the ethics committee of the Fluminense Federal University (CEUA n° 9353110221).

### 4.2. Phenotypic Analysis

All isolates were subjected to phenotypic identification tests, including observation of colony morphology and hemolysis on 5% sheep blood agar plates (Plast Labor, Rio de Janeiro, Brazil), and CAMP production and hippurate hydrolysis assays, as described elsewhere [50]. In addition, species identity was confirmed by MALDI-TOF MS [51]. The ability to ferment lactose was assessed for all GBS isolates, as previously described [50]. Briefly, bacterial colonies were inoculated into 5 mL of Heart Infusion Broth (Difco™, Becton Dickinson, Franklin Lakes, NJ, USA) containing 1% lactose (Reagen, Colombo, Brazil) and 0.1% bromocresol (Pharmacia Biotech, São Paulo, Brazil) purple as a pH indicator. Tubes were incubated for up to seven days at 37 °C and observed daily. A color change to yellow was considered indicative of lactose fermentation.

Minimum inhibitory concentrations (MICs) of antimicrobials were determined by the microdilution method, using a Sensititre Mastitis Plate™ (Thermo Fisher Scientific, Waltham, MA, USA) for isolates that harbored AMR genes based on genomic analysis (see below). MIC breakpoints have not been fully defined for bovine GBS, so data were interpreted based on human breakpoints for antimicrobials not listed for cattle [25,26]. *Streptococcus pneumoniae* ATCC 49619 was used for quality control.

### 4.3. Genomic and Phylogenetic Analysis

Genomic DNA was extracted with a DNeasy Blood & Tissue Kit (Qiagen, Hilden, Germany). Whole-genome sequencing (WGS) was conducted at the Wellcome Sanger Institute (as part of the global GBS surveillance study JUNO, https://www.gbsgen.net/) or by MicrobesNG (Birmingham, UK) using the Illumina NovaSeq platform. WGS data were used to predict capsular types and STs, and to detect the presence of AMR genes, surface protein genes, pilus island genes, and virulence genes using the GBS Typer v1.0.11 (https://github.com/sanger-bentley-group/GBS-Typer-sanger-nf). Any new sequence types that were detected were submitted to pubMLST (https://pubmlst.org) for ST assignment. STs were grouped into clonal complexes (CCs) with the minimum spanning tree algorithm using Bionumerics v7.6 software. Mobile genetic elements (MGEs) associated with AMR genes were identified using the Mobile Element Finder software available on the Centers for Genomic Epidemiology website (https://cge.food.dtu.dk/services/MobileElementFinder/, accessed on 20 September 2023), considering a query coverage of 100% and sequence identity of >98%.

To construct a core gene phylogeny, nucleotide sequences were annotated with Prokka v.1.14.6 [52] and a core gene alignment was generated by Panaroo v.1.3.0 using the sensitive mode and default parameters. All genes present in at least 95% of isolates were aligned using MAFFT version 7.471 [53,54]. IQ-TREE was used to infer a maximum likelihood tree under the GTR+F model [55]. The core gene tree figure was created using Microreact [56].

## 5. Conclusions

GBS is an important causative agent of CBM in Brazil. The bovine GBS population in Brazil is dominated by two lineages, CC91 and CC103. These lineages are evolutionarily distant, have been circulating in the country since the 1990s, became major lineages recently, and are unlikely to pose a public health risk. CC103 is associated with serotype Ia and AMR, whereas CC91 is associated with serotype III and antimicrobial susceptibility. The drivers of their emergence are unknown and may be linked to transmission rather than AMR. The control of CBM in Brazil would benefit from improved biosecurity, diagnostics, and milking time hygiene, and possibly from vaccination, with a focus on serotypes Ia and III. Antimicrobial stewardship should focus on the use of narrow-spectrum antimicrobials for mastitis treatment, notably penicillin, both because there is no resistance to this compound and because its use would reduce the risk of the selection of GBS carrying macrolide, lincosamide, or other AMR genes that could pose a risk to human health.

## Data Availability

The genomes of the isolates included in this study are available in the European Nucleotide Archive (Project: PRJEB34470) and GenBank (BioProject ID: PRJNA1086968) databases.

## Supplementary Materials

The following supporting information can be downloaded at: https://www.mdpi.com/article/10.3390/antibiotics13050389/s1, Figure S1: Frequency distribution of 156 Group B *Streptococcus* isolates from bovine milk, collected from 1987 to 2021 from 45 herds in Brazil. The figure shows the presence of six clonal complexes (CCs) and three singletons, colored as indicated in the legend on the right. CC103 isolates were first detected in 1997, while the first identification of CC91 isolates in our collection dates to 1999. Table S1: Distribution of bovine Group B *Streptococcus* isolates collected in Brazil from 1987 to 2021 according to the year of isolation, herd of origin, sequence type (ST), clonal complex (CC), capsular type, and presence of antimicrobial resistance (AMR) genes and mobile genetic elements (MGEs).
