# Supplementary material for: Long-Term Co-Circulation of Host-Specialist and Host-Generalist Lineages of Group B Streptococcus in Brazilian Dairy Cattle with Heterogeneous Antimicrobial Resistance Profiles
Source: Antibiotics (Basel). 2024 Apr 25;13(5):389. doi: 10.3390/antibiotics13050389 (PMC11117364; doi:10.3390/antibiotics13050389)
Supplement: Supplementary file 1 [file antibiotics-13-00389-s001.zip › Figure S1.pdf]

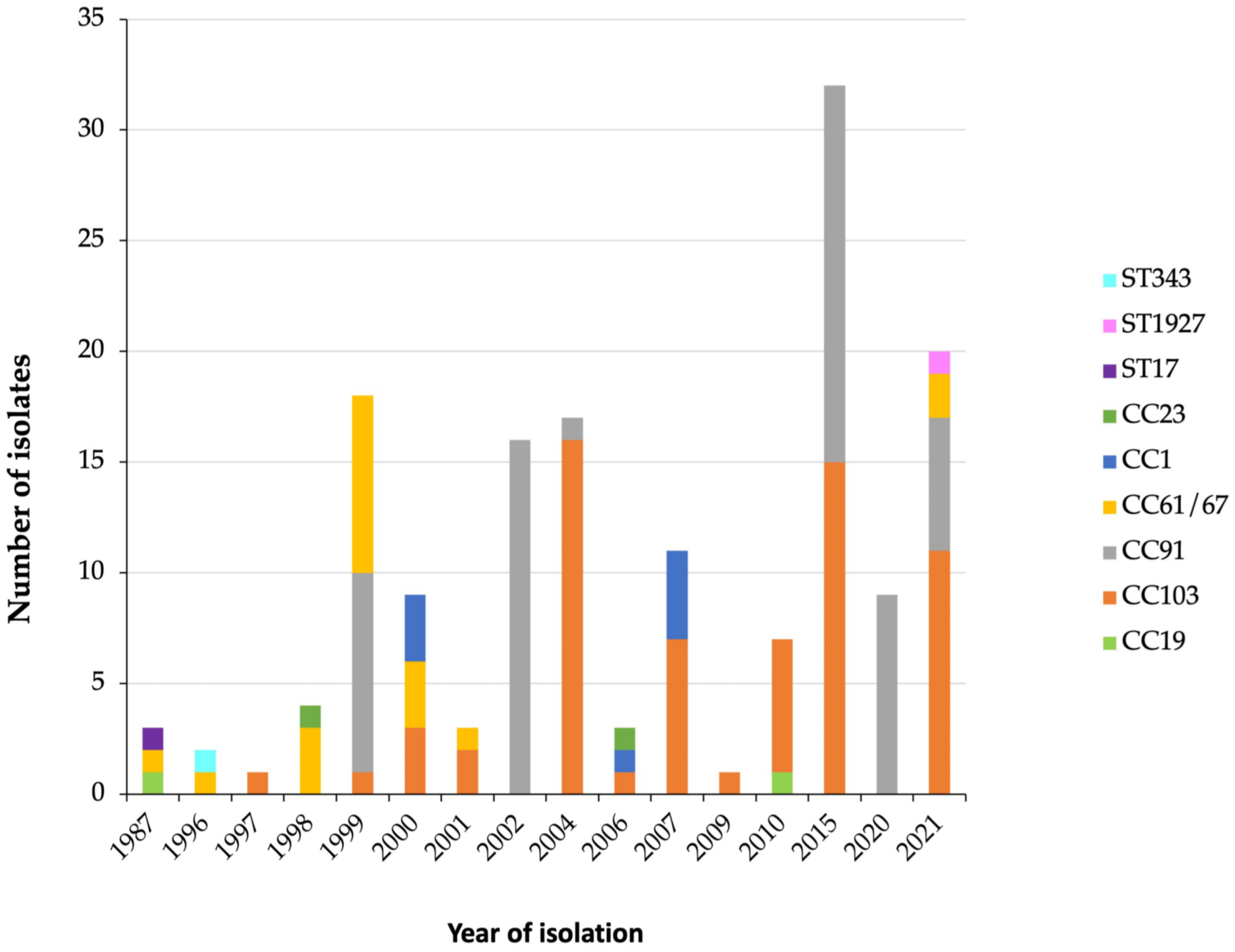

Figure S1: Frequency distribution of 156 Group B *Streptococcus* isolates from bovine milk, collected from 1987 to 2021 from 45 herds in Brazil. The figure shows the presence of six clonal complexes (CCs) and three singletons, colored as indicated in the legend on the right. CC103 isolates were first detected in 1997, while the first identification of CC91 isolates in our collection dates to 1999.
