# Supplementary material for: Long-Term Co-Circulation of Host-Specialist and Host-Generalist Lineages of Group B Streptococcus in Brazilian Dairy Cattle with Heterogeneous Antimicrobial Resistance Profiles
Source: Antibiotics (Basel). 2024 Apr 25;13(5):389. doi: 10.3390/antibiotics13050389 (PMC11117364; doi:10.3390/antibiotics13050389)
Supplement: Supplementary file 1 [file antibiotics-13-00389-s001.zip › Table S1.pdf]

Table S1. Distribution of bovine Group B *Streptococcus* isolates collected in Brazil from 1987 to 2021 according to the year of isolation, herd of origin, sequence type (ST), clonal complex (CC), capsular type, and presence of antimicrobial resistance (AMR) genes and mobile genetic elements (MGE).

Herd	Year	Total number of isolates	Number of isolates per profile	ST	CC	Capsular type	AMR genes	MGE
H1	2001	1	1	103	103	Ia	*tetM*	Tn*6009*
H2	2001	1	1	67	61/67	NT*	*aadE*, *ermB*, *tetO*	not detected
H3	2002	9	8	91	91	III	not detected	not detected
	2002		1	not determined (*glcK* atypical) - CC91	91	III	not detected	not detected
H4	2002	7	7	91	91	III	not detected	not detected
H5	2001	1	1	103	103	Ia	*tetM*	Tn*6009*
H6	2004	17	1	not determined (*glcK* atypical) - CC103	103	Ia	*tetM*	Tn*6009*
	2004		9	103	103	Ia	*tetO*	not detected
	2004		6	103	103	Ia	not detected	not detected
	2004		1	91	91	III	not detected	not detected
H7	1987	1	1	61	61/67	II	*tetM*	Tn*6009*
H8	1987	1	1	19	19	III	not detected	not detected
H9	1987	1	1	17	Singleton	III	*tetM*	not detected
H10	2015	14	11	91	91	III	not detected	not detected
	2015		1	not determined (*glcK* atypical) - CC91	91	III	not detected	not detected
	2015		2	not determined (*glcK* atypical) - CC91	91	III	*aadE*, *ermB*, *tetO*	not detected
H11	1998	5	1	not determined (*glcK* atypical) - CC61/67	61/67	III	*tetO*	not detected
	1998		3	1929	61/67	III	*tetO*	not detected
	2000		1	67	61/67	III	*aadE*, *ermB*, *tetO*	not detected
H13	1999	2	2	not determined (*glcK* atypical) - CC61/67	61/67	III	not detected	not detected
H12	1999	3	3	91	91	III	not detected	not detected
H15	1999	1	1	67	61/67	II	*aadE*, *ermB*, *tetO*	not detected
H14	2015	7	2	91	91	III	*ermB*, *tetO*	not detected
	2015		1	not determined (*glcK* atypical) - CC91	91	III	*ermB*, *tetO*	not detected

	2015		1	103	103	Ia	*aadE*, *ermB*, *tetO*	not detected
	2015		3	1918	103	Ia	*tetM*	Tn*6009*
H16	2015	11	11	103	103	Ia	*aadE*, *ermB*, *tetO*	not detected
H17	2010	8	7	103	103	Ia	*aadE*, *ermB*, *tetO*	not detected
	2010		1	19	19	V	*tetM*	not detected
H18	2021	4	2	67	61/67	II	*ermB*, *tetO*	not detected
	2021		1	91	91	III	not detected	not detected
	2020		1	not determined (*glcK* atypical) - CC91	91	III	*aadE*, *ermB*, *tetO*	not detected
H19	1996	1	1	343	Singleton	III	*aadE*, *ermB*, *tetO*	not detected
	1998	3	1	not determined (*glcK* atypical) - CC61/67	61/67	III	not detected	not detected
	1999		1	91	91	III	not detected	not detected
	1999		1	1930	61/67	III	not detected	not detected
H21	1999	5	1	103	103	Ia	*tetO*	not detected
	1999		4	1930	61/67	III	*tetM*	Tn*6009*
H22	1998	1	1	55	23	III	not detected	not detected
H23	2000	1	1	not determined (*glcK* atypical) - CC1	1	Ia	*tetO*	not detected
H24	2007	1	1	1	1	V	*aadE*, *ermB*, *tetO*	not detected
H34	2000	1	1	103	103	Ia	*tetO*	not detected
H27	2007	1	1	1	1	V	*aadE*, *ermB*, *tetO*, *tetM*	Tn*6009*
H25	2006	1	1	23	23	Ia	*tetM*	not detected
H26	2007	2	1	1	1	V	*aadE*, *ermB*, *tetO*	not detected
	2007		1	1	1	V	*aadE*, *ermB*, *tetO*, *tetM*	Tn*6009*
H28	2007	2	2	not determined (*glcK* atypical) - CC103	103	Ia	*tetM*	ISLgar5 (IS*256*)
H29	2007	1	1	314	103	Ia	*tetM*	ISLgar5 (IS*256*)
H30	2007	3	2	103	103	Ia	*tetM*	ISLgar5 (IS*256*)

	2007		1	not determined (*glcK* atypical) - CC103	103	Ia	*tetM*	ISLgar5 (IS*256*)
H31	2007	1	1	not determined (*glcK* atypical) - CC103	103	Ia	*tetM*	ISLgar5 (IS*256*)
H32	2006	1	1	not determined (*glcK* atypical) - CC103	103	Ia	*tetO*	not detected
H33	2006	1	1	1	1	V	*erm*A, *tetM*	Tn*6009*
H35	2000	2	1	196	1	IV	*tetM*	Tn*6009*
	2000		1	1	1	V	*tetM*	Tn*6009*
H36	2021	13	2	1934	91	III	not detected	not detected
	2021		11	103	103	Ia	*tetM*	not detected
H37	1996	1	1	1932	61/67	II	*aadE*, *lnu*C, *tetO*	not detected
H40	2021	4	4	not determined (*glcK* atypical) - CC91	91	III	*aadE*, *ermB*, *tetO*	not detected
H41	2021	1	1	1927	Singleton	II	*tetO*	not detected
H39	2000	1	1	not determined (*glcK* atypical) - CC103	103	Ia	*ermB*, *tetO*	not detected
H42	2020	6	5	91	91	III	not detected	not detected
	2020		1	not determined (*glcK* atypical) - CC91	91	III	not detected	not detected
H38	1999	5	5	91	91	III	not detected	not detected
H43	2021	1	1	1934	91	III	not detected	not detected
H44	1997	1	1	not determined (*glcK* atypical) - CC103	103	Ia	*ermB*, *tetO*	not detected
H45	2000	1	1	103	103	Ia	*tetO*	not detected

*NT: non typeable
